# Native Bacterial Endophytes Promote Host Growth in a Species-Specific Manner; Phytohormone Manipulations Do Not Result in Common Growth Responses

**DOI:** 10.1371/journal.pone.0002702

**Published:** 2008-07-16

**Authors:** Hoang Hoa Long, Dominik D. Schmidt, Ian T. Baldwin

**Affiliations:** Max Planck Institute for Chemical Ecology, Jena, Germany; Massachusetts General Hospital, United States of America

## Abstract

**Background:**

All plants in nature harbor a diverse community of endophytic bacteria which can positively affect host plant growth. Changes in plant growth frequently reflect alterations in phytohormone homoeostasis by plant-growth-promoting (PGP) rhizobacteria which can decrease ethylene (ET) levels enzymatically by 1-aminocyclopropane-1-carboxylate (ACC) deaminase or produce indole acetic acid (IAA). Whether these common PGP mechanisms work similarly for different plant species has not been rigorously tested.

**Methodology/ Principal Findings:**

We isolated bacterial endophytes from field-grown *Solanum nigrum*; characterized PGP traits (ACC deaminase activity, IAA production, phosphate solubilization and seedling colonization); and determined their effects on their host, *S. nigrum,* as well as on another Solanaceous native plant, *Nicotiana attenuata*. In *S. nigrum*, a majority of isolates that promoted root growth were associated with ACC deaminase activity and IAA production. However, in *N. attenuata*, IAA but not ACC deaminase activity was associated with root growth. Inoculating *N. attenuata* and *S. nigrum* with known PGP bacteria from a culture collection (DSMZ) reinforced the conclusion that the PGP effects are not highly conserved.

**Conclusions/ Significance:**

We conclude that natural endophytic bacteria with PGP traits do not have general and predictable effects on the growth and fitness of all host plants, although the underlying mechanisms are conserved.

## Introduction

Symbiotic interactions are the driving force in ecosystems; symbiosis ranges from parasitism to mutualism and includes everything in between. The fitness outcomes for plants differ accordingly: if a plant is highly susceptible to pathogens, its fitness is likely to be low in pathogen-rich environments; if a plant cooperates with mutualists, it is likely to thrive even in adverse environments. Bacteria, which colonize the interface between living plant roots and soil, namely the rhizosphere, are abundant symbiotic partners of plants. These so-called rhizobacteria are said to be plant growth promoting (PGP). Those microbes able to colonize plant roots internally without negatively affecting the host are called endophytes [1]. Although all of the approximately 300,000 plant species have been estimated to harbor one or more endophytes [2], few relationships between plants and these endophytes have been studied in detail; the legume-rhizobia symbiosis is an exception. The mutualistic interaction of legumes with rhizobia involves finely tuned recognition steps which ultimately lead to the production of root nodules in which the plants accommodate the bacteria [3]. For other endophytic rhizobacteria, the processes of host-microbe signaling and colonization, and the mechanisms leading to mutual benefit are less-well characterized.

Bacterial endophytes can accelerate seedling emergence, promote plant establishment under adverse conditions and enhance plant growth [4], [5]. Endophytic bacteria are believed to elicit plant growth promotion in one of two ways: either (1) indirectly by helping plants acquire nutrients, e.g. via nitrogen fixation, phosphate solubilization [6] or iron chelation [7], by preventing pathogen infections via antifungal or antibacterial agents, by outcompeting pathogens for nutrients by siderophore production, or by establishing the plant's systemic resistance [8]; or (2) directly by producing phytohormones such as auxin or cytokinin [9], or by producing the enzyme 1-aminocyclopropane-1-carboxylate (ACC) deaminase, which lowers plant ethylene levels [10]. In addition to these plant-growth-promoting traits, endophytic bacteria must also be compatible with host plants and able to colonize the tissues of the host plants without being recognized as pathogens [11]. A particular bacterium may affect plant growth and development using one or more of these mechanisms, and may use different ones at various times during the life cycle of the plant. While the mechanisms of growth promotion appear to be universal–for example, by changing a plant's phytohormone metabolism–it remains unclear how consistently bacterial endophytes elicit responses in host and non-host plant species.

Many studies have documented the interaction between PGP rhizobacteria and host plants. A mechanistic model was previously developed by Glick et al. [12] to explain the role of bacterial ACC deaminase and IAA in promoting plant growth. Ethylene and IAA are implicated in virtually all aspects of plant growth and development, ranging from seed germination to shoot growth and leaf abscission [13]. Therefore, production of ACC deaminase and IAA is likely an important and efficient way for endophytes to manipulate their plant hosts. Endophytic bacteria containing ACC deaminase promoting plant growth are usually located inside plant roots in the apoplast. The cleavage of ACC results in ammonia and α-ketobutyrate which are readily metabolized by the bacteria. In this way, these bacteria act as a sink for ACC. By lowering ET levels, the bacteria increase the growth of plant roots and shoots and reduce the inhibitory effects of ethylene synthesis. In addition to being produced by plants, IAA is also produced by root-associated bacteria such as *Enterobacter* spp., *Pseudomonas* spp., and *Azospirillium* spp. [14]. Lowering ethylene in plant roots also relieves the suppression of auxin response factor synthesis, and indirectly increases plant growth [15].

The central role of phytohormone signaling in plant-endophyte interactions suggests two scenarios: (1) Endophytic bacteria with general PGP traits, such as the ability to produce IAA and ACC deaminase, promote growth uniformly across plant species including non-hosts [16], [17]. Such endophytes are expected to be readily recruited by a novel host. (2) Once recruited by a particular host, endophytes undergo host-specific adaptations; the upshot is a highly specialized, finely tuned mutualism. Such mutualisms may make plants better able to tolerate the endophyte and the endophyte in turn more responsive to the plant's metabolism [1]. Hence, non-host plants might recognize these endophytes as pathogens despite their plant-growth-promoting properties, either because they are pathogens for the non-host or because they elicit inappropriate responses in a non-host-plant species [18].

In order to test these two hypotheses, we first isolated and identified plant-growth-promoting endophytic bacteria from black nightshade (*Solanum nigrum)*, a native plant that interacts with many partners in its habitat [19]. We then selected the isolates exhibiting the clearest plant-growth-promoting traits and exerting the strongest positive effects on root growth of *S. nigrum;* we inoculated a closely related plant species, *Nicotiana attenuata,* with these isolates. In addition, bacterial type strains from a culture collection with known PGP traits were analyzed to determine whether their general PGP effects translate to fitness benefits in *N. attenuata* and *S. nigrum*. We report markedly different growth and fitness responses of these plant species to the same bacterial strains. Our results are consistent with the scenario in which plant growth promotion by native endophytic bacteria is highly species-specific, regardless of whether or not they express general PGP traits.

## Materials and Methods

### Plant materials

The following inbred lines were used in all experiments: *S. nigrum* Sn30 [19]; *N. attenuata* (synonymous with *N. torreyana*) genotype Utah [20]. Seed germination procedures of *S. nigrum* and *N. attenuata* are described elsewhere [19], [21].

### Bacterial type strains

Six bacterial species were selected from the German culture collection (DSMZ) *Pseudomonas brassicacearum* D13227, *Bacillus pumilis* D1794, *Pseudomonas putida* D50194, *Pseudomonas marginalis* D50276, *Methylobacterium fujisawaense* D5686 and *Pseudomonas fluorescens* D8568.

### Isolation of culturable endophytic bacteria

*S. nigrum* plants were individually collected from field sites near Dornburg, Germany, or near the Max Planck Institute for Biogeochemistry, Jena, Germany. Roots were washed in tap water to remove soil; leaves, stems and roots were separated. Roots of *S. nigrum* plants growing in the margins of agricultural fields in the Dornburg and Saale valley were similarly collected. Endophytic bacteria were isolated as described by Long et al. [22]. Briefly, endophytic bacteria were isolated after removing epiphytes by surface disinfection using serial washing in 70% ethanol for 1 min, sodium hypochlorite solution (3% available Cl^−^) for 3 min and three rinses in sterilized distilled water. The disinfection process was checked by plating aliquots of the sterile distilled water used in the final rinse onto 0.5x YPDA (Sigma, Steinheim, Germany) and incubating the plates at 30°C for 2–10 days. After surface disinfection, the leaf, stem or root tissue was cut and titrated in distilled water; appropriate dilutions were plated onto 0.5x YPDA and incubated at 30°C for 2–10 days. After incubation, colonies were picked from the plates, inoculated on 0.5x YPDA slant tubes, incubated at 30°C for 2 days and stored at 4°C. Each culture was suspended in 20% glycerol solution and stored at −80°C for long-term use.

### Plant culture

Seeds were surface-sterilized as described by Schmidt et al.[19]. Bacterial suspensions in sterile distilled water (10^8^cfu ml^−1^) were used for seed inoculation; control seeds were treated with sterile distilled water only. The inoculated seeds (20–30 seeds) were incubated at room temperature overnight and transferred onto sterile filter papers (Whatman No.1) in Petri dishes. One week after bacterial inoculation, root and hypocotyl lengths were measured. Two independent experiments were carried out for all seedling assays.

### Characterization of PGP traits of endophytic bacteria

Production of ACC deaminase was determined as described by Glick et al. [10], who measured the amount of a-ketobutyrate produced when the enzyme ACC deaminase cleaves ACC. The nmoles of α-ketobutyrate produced by this reaction was determined by comparing the absorbance at 540 nm of a sample to a standard curve of α-ketobutyrate ranging between 0.1 and 1.0 nmol. IAA production was determined as described by Bric et al. [23] by the colormetric method. Phosphate solubilization was determined as described by Verma [24]. Seedling colonization was carried out by inoculating surface sterilized seeds with bacteria and re-isolating bacteria from roots after 7 days of growth.

### Identification of endophytic bacterial isolates by 16S rRNA gene sequencing

Total bacterial DNA was isolated from 1-day-old cultures on agar plates. Single colonies were resuspended to obtain suspensions of approximately 10^5^cfu ml^−1^. 0.5 µl of suspension was mixed with 4.5 µl extraction buffer (10 mM Tris-HCl pH 7.6; 50 mM KCl; 0.1% Tween 20). The mixture was heated at 100°C for 10 min and immediately placed on ice. After centrifugation at 6000*xg* for 5 min, the supernatant was used for PCR. Amplification of 16S rDNA was performed in a 10 µl final volume containing 1 µl of total DNA, 10 µM of primer F27 (5′-AGAGTTTATCMTGGCTCAG-3′) and R1492 (5′-GRTACCTTGTTACGACTT-3′) [25], 10 mM of each dNTP, 5 mM MgCl_2_ and 0.05U of Taq DNA polymerase (Eppendorf, Hamburg, Germany). A negative control (PCR mixture without DNA template) was included in all PCR experiments. The reaction conditions were as follows: 95°C for 2 min followed by 30 cycles of denaturation at 95°C for 15 s, annealing at 55°C for 20 s and primer extension at 72°C for 1 min, followed by a final extension at 72°C for 5 min. The reaction products were separated by running the PCR mixture in 1.2% (w/v) agarose containing ethidium bromide. For sequencing, PCR products were purified using QIAquick™ Gel Extraction Kit (QIAGEN, Hilden, Germany) following the manufacturer's manual. Direct sequencing using the same primers was conducted in Big Dye Mix (Applied Biosystems, Foster City, CA 94404, USA) and purification of sequencing reactions was performed using NucleoSEQ kit (Macherey-Nagel, Duren, Germany). Analysis of sequences was carried out with basic sequence alignment BLAST program run against the database from National Center for Biotechnology Information Blast (www.ncbi.nlm.nih.gov/BLAST).

### Seedling vigor assay

Seventy-seven isolates were used for seed treatment. After surface disinfection, *S. nigrum* seeds were treated with pure cultures of these isolates (10^8^cfu ml^−1^) in distilled water for 24 h; control seeds were incubated in sterile distilled water for 24 h.

Germination tests were carried out by the paper towel method [26]. The germination paper was soaked in distilled water, 15–20 bacterially treated seeds and untreated seeds were placed on paper towels, rolled and wrapped with polythene to prevent drying, and incubated at 25±2°C for seven days, when the towels were unrolled and the number of seeds that had germinated was counted. On the same day, seedling vigor was analyzed using the method of Abdul Baki and Anderson [27]. The lengths of roots and hypocotyls of all the individual seedlings were measured. The vigor index (VI) was calculated using the formula VI  =  (mean root length + mean hypocotyl length)*% germination. The experiment was repeated twice. The strains which gave high germination and vigor were selected for further experiments.

### Transformation of bacteria with pDSK-GFPuv plasmid

Preparation of electro-competent cells was carried out as standard protocol for *E. coli* with some modifications. Briefly, 0.5 l YPD broth (Sigma, Steinheim, Germany) was inoculated with 5 ml overnight, cultured and grown to an OD_600_ of 0.5–0.7 (0.5xYPD broth; 30°C ; 220 rpm). Cells were harvested by centrifugation (8000×*g*, 4°C) and washed 4 times in ice-cold 10% glycerol. Finally, the bacterial pellet was resuspended in 1.5 ml 10% glycerol, divided into 40 µl aliquots, and stored at −80°C. Transformation of bacteria with pDSK-GFPuv plasmid was done by electroporation as described by Wang et al. [28]. Fluorescent transformants containing the plasmid pDSK-GFPuv were selected on LB agar plates supplemented with 50 µg ml^−1^ kanamycin and identified under long-range UV light (365 nm).

### Confocal laser-scanning microscopy (CLSM)

Seeds were inoculated with GFP-labeled bacteria as described above. Seven days after inoculation, root colonization was observed with a CLS microscope LSM510 (Carl Zeiss, Jena, Germany) equipped with an Argon laser (458, 477, 488, 514 nm) and detectors for monitoring GFP (495–590 nm). Images were collected in a z-series from 30 to 130 optical sections ranging from 1.3 to 7.2 µm in thickness. Optical sections, maximum intensity projections and overlays were generated, and single images were processed by selecting a subset from a z-series using the Zeiss LSM Image Browser, version 4.0 (Carl Zeiss).

### Seedling ethylene measurement

Ethylene emissions from seedlings were measured continuously and non-invasively in real-time with a photoacoustic spectrometer (INVIVO, Saint Ausgustin, Germany) as described by von Dahl et al. [29]. Inoculated seeds that had germinated in 100 ml cuvettes for 7 days at 25±2°C were subjected to ethylene measurements. Five cuvettes were used for one treatment and empty cuvettes as well as cuvettes with seeds treated with sterile distilled water served as controls.

### Data analysis

Analysis of the data was carried out using StatView software package (SAS Institute) with a completely randomized analysis of variance (P<0.05). The Fisher's PLSD test was used to compare means of root length and hypocotyl length of seedlings, stalk length, capsule number per plant and fruit number per plant in all experiments. Simple regression analysis was used to compare relationships between ACC deaminase activity and root length, ACC deaminase activity and ethylene measurement, and bacterial IAA and root length.

## Results

### Isolation and characterization of endophytic bacteria from *S. nigrum*

Seventy-seven endophytic bacterial isolates were isolated from roots, stems and leaves of black nightshade plants (*S. nigrum*) grown in two different native habitats in Jena, Germany. They were all characterized for their ability to 1) produce ACC deaminase; 2) synthesize the phytohormone IAA; 3) solubilize phosphate; and 4) colonize seedlings, since these traits are associated with plant growth promotion [30]. Twenty-three isolates were able to grow on the minimum medium DF salt supplemented with ACC as a sole N source, suggesting that they have ACC deaminase activity. One isolate was able to produce IAA without supplementation of Trp and 28 were able to produce IAA with supplementation of Trp. Six isolates were able to solubilize inorganic phosphate. Twenty-four isolates were able to colonize *S. nigrum* seedlings internally (Table 1).

**Table 1:** Biochemical characteristics of endophytic bacteria isolated from *S. nigrum*.

Origin*	No. isolates	Growth on DF salt with ACC†	*In vitro* IAA production‡		Phosphate solubilization	Seedling colonization
			−Trp	+Trp		
BGCR1	11	1	0	3	0	2
BGCSL1	4	0	0	1	0	0
BGCR2	13	5	0	5	2	5
BGCSL2	13	1	0	2	1	2
DR	9	2	1	5	1	3
DSL	8	5	0	5	0	4
DSR	12	2	0	2	1	2
SSR	7	6	0	5	1	6
**Total**	**77**	**23**	**1**	**28**	**6**	**24**

* Isolation of endophytic bacteria from roots/stem leaves from plants collected in 2 field plots of Max Planck Institute for Biogeochemistry (BGCR/SL), roots/stem leaves from plants collected in the Dornburg field (DR/SL), roots from plants grown in Dornburg field soil in greenhouse (DSR) and roots from plants grown in the greenhouse in soil from the Saale (SSR);

† ACC: 1-Aminocyclopropane-1-carboxylate;

‡ IAA: Indole-3-acetic acid; Trp: DL-Tryptophan

### Screening endophytic bacteria for plant growth promotion

**Figure 1:**
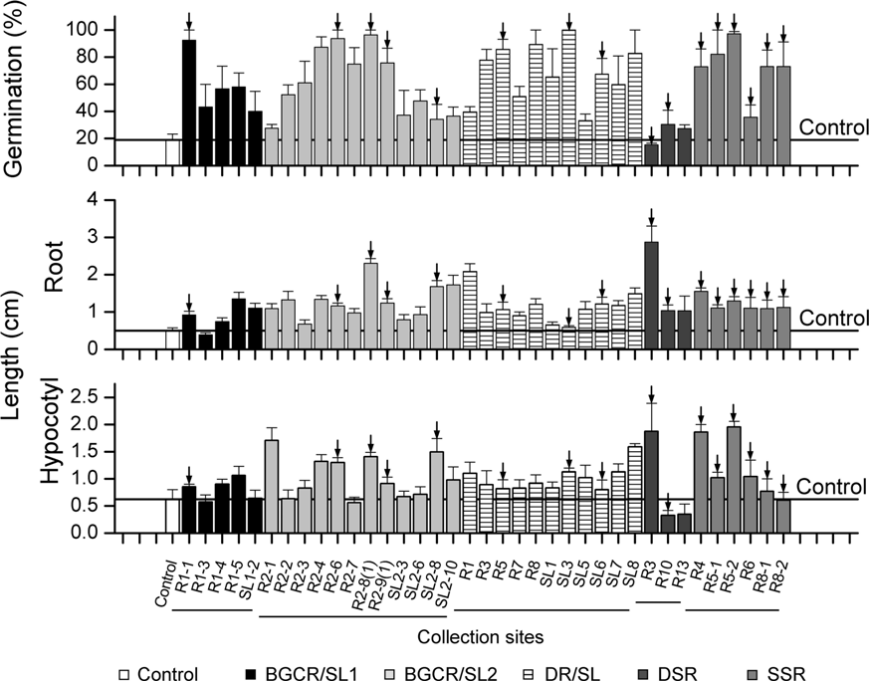
Effects of endophytic bacteria on seedling vigor. Mean (±SE; n  =  30–40) percentage germination, root length (cm) and hypocotyl length (cm). Seeds treated with sterile distilled water served as controls (white bars). The different shadings of the bars indicate the origin of the isolate (roots/stem leaves from *S. nigrum* plants collected from 2 field plots of Max Planck Institute for Biogeochemistry (BGCR/SL), roots/stem leaves from *S. nigrum* plants collected in the Dornburg field (DR/SL), roots from *S. nigrum* plants grown in Dornburg field soil in the glasshouse (DSR) and roots from plants grown in the glasshouse in soil from the Saale valley (SSR)). Arrows identify the sixteen isolates that were selected for further study.

A *S. nigrum* seedling vigor assay was used to screen the endophytic bacterial isolates for their PGP ability, using the isolates' effects on seed germination, root and hypocotyl growth; 37 of 77 isolates increased seedling vigor in the first assay and were screened a second time (Fig. 1). Of these 37 isolates, 22 significantly enhanced seed germination–up to 100%–compared with untreated controls (Fisher's PLSD test; *P*<0.05). One isolate, DSR3, inhibited seed germination. Twenty-seven isolates significantly increased the seedling root length compared with the control (Fisher's PLSD test; *P*<0.05). Eleven isolates significantly promoted the hypocotyl growth of seedlings (Fisher's PLSD test; *P*<0.05). Four isolates inhibited either root or hypocotyl growth (Fig. 1). Sixteen isolates were selected for further study because they had 1) one or more of the PGP traits (Table 1) and 2) enhanced seedling growth in both screening trials. Isolate DSR10 strongly inhibited seedling growth and was used as a negative control in further experiments.

### Identification of bacterial isolates

Sixteen isolates were selected based on their PGP traits and seedling growth promotion. The 16S rRNA gene was amplified in these isolates using universal primers, and sequenced. The sequences were similar to those of 6 bacterial genera, namely *Pseudomonas, Acinetobacter, Pantoea* (formerly *Enterobacter*), *Agrobacterium*, and *Aeromonas* (Table 2) with high homology hits in the database ranging from 95 to 100% similarity. Ten isolates were identified to species. Isolate DSR10 was identified as *Agrobacterium tumefaciens*, a phytopathogen. The sequences are deposited in GenBank (http://www.ncbi.nlm.nih.gov/Genbank/) under the accession numbers shown in Table 2.

**Table 2:** Identification of bacterial isolates using 16S rRNA gene sequences.

Bacterial isolates	GenBank accession number	Closest match according to the 16S rRNA gene sequence	No. of bases	Max. score	% match
BGCR1-1	EU434624	*Enterobacter agglomerans* strain A17	775	1400	99
BGCR2-6	EU434628	*Pseudomonas* sp. BSs20166	682	1205	98
BGCR2-8(1)	EU434629	*Pseudomonas* sp. S8-130	799	1476	100
BGCR2-9(1)	EU434630	*Pseudomonas brassicacearum* isolate MA250	868	1604	100
BGCSL2-8	EU434635	*Pseudomonas lutea* strain PSB2	806	1290	95
DR5	EU434637	*Pseudomonas thivervalensis* strain H2P3	506	922	99
DSL3	EU434639	*Enterobacter agglomerans* strain A17	888	1583	98
DSL6	EU434640	*Pantoea agglomerans* strain PTA-AF1	661	1216	99
DSR3	EU434642	*Aeromonas veronii* strain 211c	790	1448	99
DSR10	EU434641	*Agrobacterium tumefaciens* strain CCBAU 85035	636	994	95
SSR4	EU434643	*Pseudomonas* sp. S8-130	910	1676	99
SSR5-1	EU434644	*Pseudomonas* sp. S8-130	902	1642	99
SSR5-2	EU434645	*Pseudomonas* sp. S8-130	763	1410	100
SSR6	EU780008	*Acinetobacter calcoaceticus* strain M10	875	1616	100
SSR8-1	EU434646	*Pseudomonas fluorescens* 16S rRNA gene, strain F113	630	1164	100
SSR8-2	EU434647	*Pseudomonas* sp. OCY4	557	1022	99

### Effects of ACC deaminase and IAA from endophytic bacteria on seedling root growth

**Figure 2:**
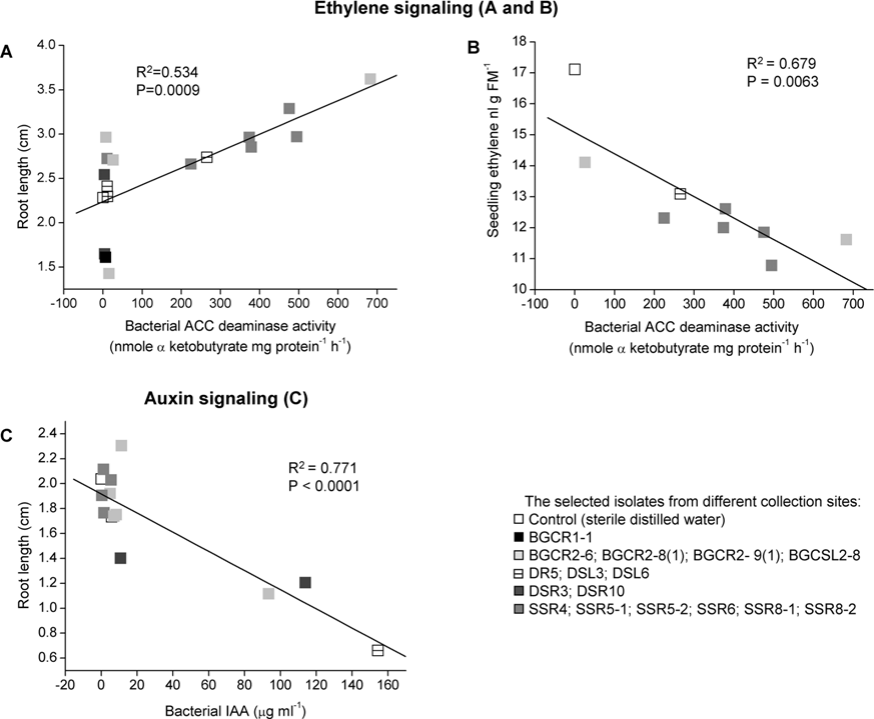
Regression of bacterial traits that influence ethylene and auxin signaling against *S. nigrum* root growth as measured in the 16 isolates identified in Figure 1. (A) Regression of bacterial ACC deaminase activity and root lengths of seedlings inoculated with bacterial isolates. (B) Regression of bacterial ACC deaminase activity and ethylene emission from seedlings inoculated with bacterial isolates. (C) Regression of bacterial IAA and root lengths of seedlings inoculated with bacterial isolates.

In order to establish a link between bacterial and plant traits, we analyzed the correlation between physiological properties of the bacterial endophytes and their effects on inoculated *S. nigrum* seedlings. Two major bacterial characteristics were addressed, namely the abilities to degrade ACC through ACC deaminase and to synthesize IAA. Of 16 selected isolates, 7 possessed high levels of ACC deaminase ranging from 200 to 700 nmol mg protein^−1^ h^−1^ and significantly enhanced root growth compared with the control (Fisher's PLSD test, *P*<0.05). In order to confirm the correlation between ACC deaminase activity and seedling root growth, we performed a regression analysis of bacterial ACC deaminase activity and the root length of seedlings that had been inoculated with the corresponding isolate. A statistically significant, positive relationship (*r* ^2^ = 0.534; *P* = 0.0009) was observed between ACC deaminase activity and root growth (Fig. 2A). In order to test whether reduced ACC levels in a plant affected ethylene metabolism, we determined the relation between bacterial ACC deaminase activity and plant ethylene emissions, using simple regression analysis. Although we found a significantly negative relationship (*r* ^2^ = 0.679 and *P* = 0.0063) between these two factors (Fig. 2B), ACC deaminase activity and subsequent lower seedling ethylene emissions did not account for all positive effects on root growth: another group of isolates with little ACC deaminase activity also promoted root growth (Fig. 2A). In addition to ACC deaminase, some isolates produce IAA (Table 1). Exogenously applying IAA to *S. nigrum* seeds has a dosage-dependent effect: IAA when added in the range of 100 µg ml^−1^ to 10 mg ml^−1^ to seeds inhibited seedling root growth, but not when added at two lower concentrations: 1 and 10 µg ml^−1^ (Fig. S1). Applying IAA (1 µg ml^−1^) to seeds significantly increased the root growth of seedlings compared with the control. Inoculating seeds with 14 different IAA-producing isolates also modified root growth. Of these, two isolates SSR5-2 and BGCR2-9(1) increased root length in the range between 1.1 and 11 µg ml^−1^ of IAA. In addition, three isolates (BGCR2-6, DSL6 and DSR10) whose IAA levels ranged from 93 to 154 µg ml^−1^ inhibited root growth. The mean value of bacterial IAA in culture and root length of seedling inoculated with the respective IAA-producing isolates was analyzed using simple regression, and we found a statistically significant negative relationship (*r* ^2^ = 0.771 and *P*<0.0001) between bacterial IAA production and root growth (Fig. 2C).

### Endophytic bacterial colonization in root

**Figure 3:**
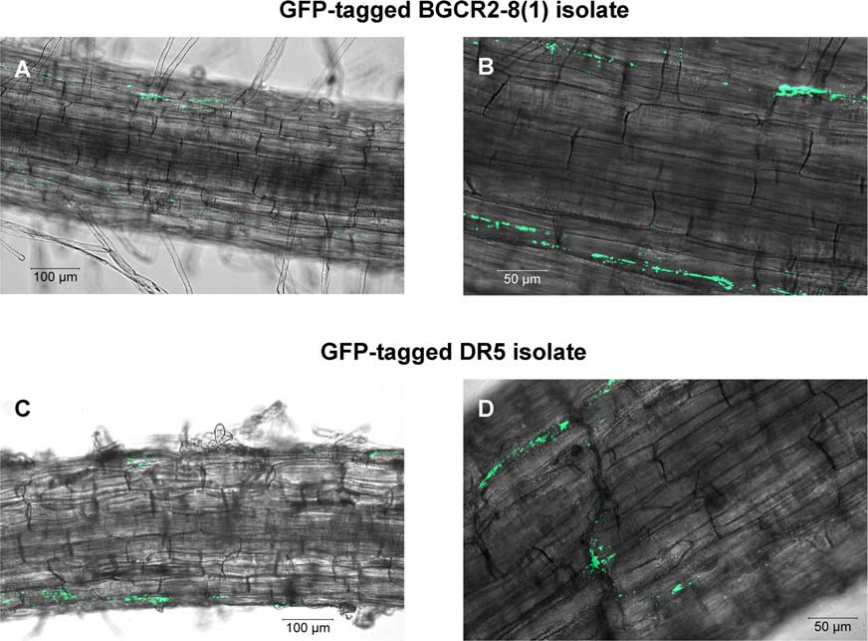
Confocal laser scanning microscopy of roots colonized by the GFP-tagged endophytic bacterial isolates. (A and B) Root colonization by GFP-tagged BGCR2-8(1) isolate at magnification of 100x (A) and 200x (B) and (C and D) root colonization by GFP-tagged DR5 isolate at magnification of 100x (C) and 200x (D).

In order to quantify the colonization, we selected seven bacterial isolates with PGP effects. All were able to colonize the inner tissues of seedlings in concentrations of up to 10^6^cfu g^−1^ FM (Table S1). GFP-tagged strains, BGCR2-8(1) and DR5, revealed that they mainly colonize cortex cells and live intercellularly (Fig. 3).

### Growth response of *S. nigrum* and *N. attenuata* to natural endophytic bacteria from *S. nigrum* and to type strains

**Figure 4:**
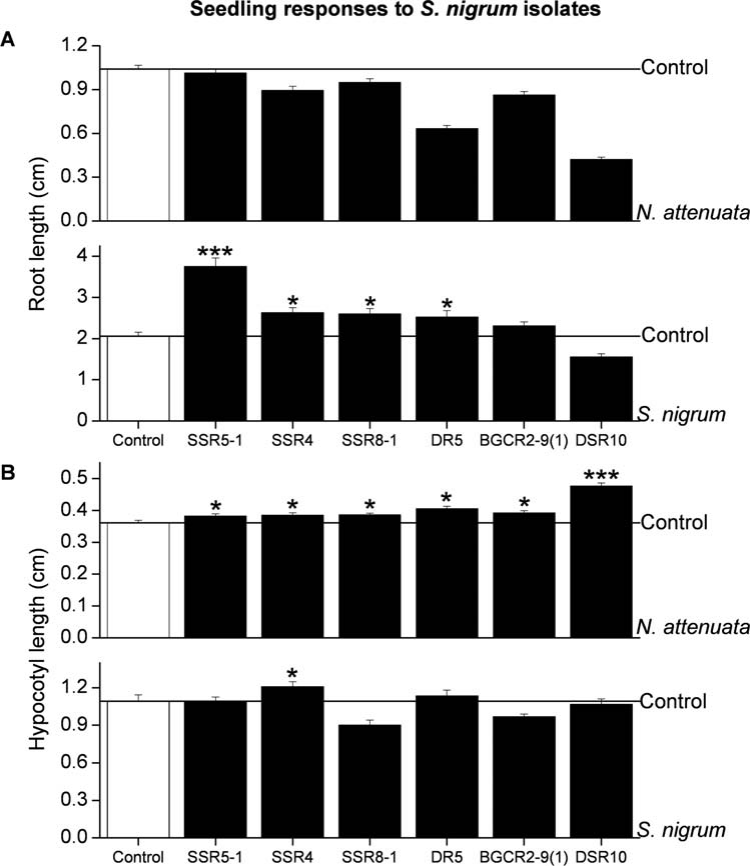
Comparison of of *S. nigrum* and *N. attenuata* seedling growth to bacterial colonization (A and B) of endophytic bacteria isolated from *S. nigrum*. Six isolates were selected based on their effects on *S. nigrum* seedling growth. (A) Mean root length (±SE) and (B) mean hypocotyl length (±SE) of *S. nigrum* and *N. attenuata* seedlings. Asterisks indicate significant differences in promotion of root and hypocotyl growth in *S. nigrum* and *N. attenuata* seedlings by the bacterial isolates compared to the control at *P*<0.05 (*); *P*<0.001 (**); and *P*<0.0001 (***).

In order to determine the growth and fitness response of host and non-host plant species to these natural endophytic bacteria, we inoculated the seeds of *S. nigrum* and *N. attenuata* with the endophytic bacterial isolates from *S. nigrum* and measured length of seedling root and hypocotyl. Six isolates from the roots of *S. nigrum* with positive, neutral and negative effects on the root growth of *S. nigrum* were selected to determine the growth response of *N. attenuata* seedlings. These two solanaceous plant species responded differently to being inoculated with these isolates. Four (SSR5-1, SSR4, SSR8-1 and DR5) significantly promoted root growth of *S. nigrum* seedlings 7 days after inoculation (Fisher's PLSD test, *P*<0.0001, *P* = 0.002, *P* = 0.004 and *P* = 0.02, respectively, Fig. 4A). However, none of the selected isolates promoted root growth in *N. attenuata* seedling and some of these isolates even inhibited root growth. These isolates had no effect on the hypocotyl growth of *S. nigrum* seedlings except for isolate SSR4, which significantly increased hypocotyl length 7 days after inoculation (Fisher's PLSD test, P = 0.0451). Most of them promoted hypocotyl length of *N. attenuata* seedlings (Fig. 4B).

**Figure 5:**
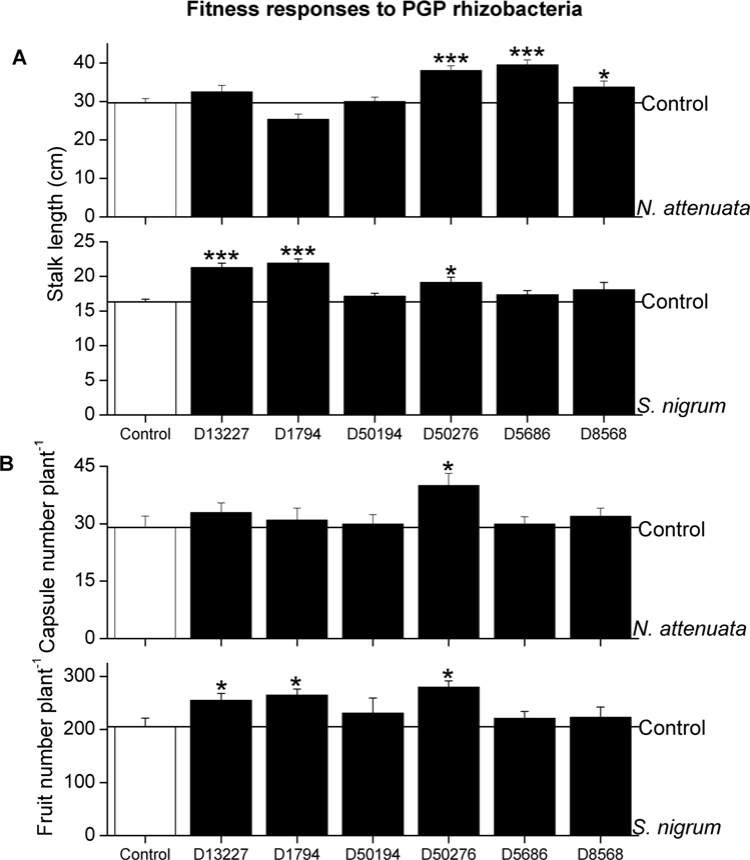
Reproductive growth and fitness responses of *S. nigrum* and *N. attenuata* plants to known mutualistic bacterial strains (*Pseudomonas brassicacearum* D13227, *Bacillus pumilis* D1794, *Pseudomonas putida* D50194, *Pseudomonas marginalis* D50276, *Methylobacterium fujisawaense* D5686 and *Pseudomonas fluorescens* D8568). (A) Mean stalk length (±SE) of *S. nigrum* and *N. attenuata*, (B) Mean (±SE) fruit number per plant (*S. nigrum*) and capsule number per plant (*N. attenuata*). Asterisks indicate significant differences (Fisher's PLSD test; *P<*0.05 (*); *P<*0.001 (**); and *P<*0.0001 (***)).

In order to test the specific response of these two Solanaceous species, we selected six bacterial species from the German culture collection (DSMZ) based on their ability to promote growth [9]. Of these six strains, four (*P. brassicacearum* D13227, *P. marginalis* D50276, *M. fujisawaense* D5686 and *P. fluorescens* D8568) exhibited ACC deaminase activity (data not shown). Three strains, *P. brassicacearum* D13227, *B. pumilis* D1794 and *P. marginalis* D50276, significantly promoted the shoot growth of *S. nigrum* 16 days after inoculation (Fisher's PLSD test, *P*<0.0001, *P*<0.0001 and *P* = 0.0044, respectively, Fig. 5A). On the other hand, the three strains, *P. marginalis* D50276, *M. fujisawaense* D5686 and *P. fluorescens* D8568, promoted shoot growth of *N. attenuata* 17 days after inoculation (Fisher's PLSD test, P<0.0001, P<0.0001 and P = 0.0311, respectively). Three strains, *P. brassicacearum* D13227, *B. pumilis* D1794 and *P. marginalis* D50276, significantly increased the fruit number of *S. nigrum* 48 days after inoculation (Fisher's PLSD test, *P* = 0.0485, *P* = 0.0183 and *P* = 0.0039, respectively), but only one of these strains, *P. marginalis* D50276, significantly enhanced the capsule production in *N. attenuata* 68 days after inoculation (Fisher's PLSD test, *P* = 0.003) (Fig. 5B). Finally, only one strain, *P. marginalis* D50276, positively affected the shoot growth and fitness of both plant species.

## Discussion

The rhizosphere is where plant roots come in contact with soil-borne microbial communities. Plant-microbe interactions mostly involve microorganisms colonizing the roots of their hosts, namely growing plants. Colonization can take the form of many different interactions ranging from symbiosis to parasitism; each interaction affects plant fitness differently. Although plant-pathogen interactions are well studied, our understanding of the complex interactions of native endophytic bacteria with their plant hosts is rudimentary, at best. We isolated bacteria from field-grown *S. nigrum* and discovered a rich endophytic community with strong prevalence of *Pseudomonas* which is well-known for plant growth promotion [31]; many of the isolated bacteria promoted growth and fitness of their host by modulating ethylene and IAA homeostasis. Although these phytohormonal pathways are conserved across plant species, the effects on a related solanaceous non-host plant, *N. attenuata*, differed, notwithstanding the similar extent to which the bacteria colonized the roots. This raises the questions: How consistent are interactions between plants and endophytic bacteria? Which mechanisms underlie these interactions? And which factors determine the outcome of the interaction?

PGP mechanisms of endophytic bacteria are thought to be similar to those of PGP rhizobacteria; namely, they affect plant growth by producing phytohormones, such as cytokinins or auxins, or by degrading hormone precursors, such as ACC by ACC deaminase [9], [12]. This is largely supported by our findings. Changes in root growth of *S. nigrum* are clearly correlated to the production of IAA and ACC deaminase by a majority of endophytic bacteria we isolated (Fig. 2). Seedling ethylene emission were significantly lower after inoculation with ACC deaminase-producing isolates and subsequently, their roots grew longer than those of untreated seedlings (Fig. 2A & B). The relatively widespread production of IAA by plant-associated bacteria suggests that bacterial IAA stimulates root the development of host plants [32], [33]. We also observed that IAA-producing isolates stimulated root growth, but only when they released low quantities of IAA; high levels of bacterial or exogenously applied IAA repressed it (Fig. 2C; Fig. S1). The concentration of exogenous IAA apparently determines the outcome of the interaction with IAA-producing endophytes.

Plant-growth-promoting rhizobacteria living in the rhizosphere are generally believed to be beneficial for all plant species they associate with because of their conserved influence of phytohormones on plant growth [16], [17], [30]. Studies on conifers and PGP rhizobacteria suggest that bacteria isolated from the rhizosphere of spruce sometimes interact with only certain ecotypes and the outcome of the interaction depends largely on experimental conditions [34]. For endophytic bacteria, even less is known. Zinniel et al. [35] studied the host range of 29 endophytic bacteria that had been isolated from sorghum or corn; 26 were able to colonize at least one other host plant in sufficient densities, leading to the conclusion that these interactions are largely unspecific. When *S. nigrum* interacts with PGP native endophytes, their influence on the homoeostasis of IAA and ethylene explains at least part of the observed phenotypes, including growth modulation. Given that hormonal regulation is conserved among plants, we had anticipated that these PGP effects of IAA and ethylene would be similar in *N. attenuata*. However, this was not the case; ACC deaminase and IAA apparently affect root growth in a highly host species-specific manner (Fig. 4A) and this specificity is determined by the bacteria.

One possible explanation for the discrepancy is the relationship between bacterial ACC deaminase and IAA, and these bacteria's mutual effects on root growth; some models describe how ACC deaminase counteracts ethylene-repressed auxin-response factors (ARFs) involved in root growth [15]. The presence of ACC deaminase-producing rhizobacteria in the rhizosphere can depress the expression of auxin response genes in the shoots [15]. Although it is well known that IAA can activate the transcription of ACC synthase [36], it is less known whether ethylene inhibits IAA transport and signal transduction [37]. The feedback loop of ethylene inhibition of IAA synthesis may limit the amount of ACC synthase, ACC, and, ultimately, ethylene that is released in response to stressful events in the life of the plant. The cross-talk between ethylene and IAA is so tightly regulated that phytohormonal imbalances might disturb plant growth and plants are generally very sensitive to IAA. Another host's endophyte might thus produce too little or too much of it and, consequently, profoundly influence plant growth. Consistent with this scenario, is the observation that *N. attenuata* root growth decreased rather than increased when exposure to some PGP bacteria (Fig. 4A). Finally, it remains to be elucidated which additional compounds are important in mediating the interaction of beneficial endophytic bacteria with *S. nigrum*.

The different responses of host and non-host species to the natural endophytic bacteria may result from a combination of several factors. The colonization success of PGP rhizobacteria reportedly increases the growth and fitness of many host plant species [34], [38]. We found no significant differences in how successfully endophytic bacteria colonize the host, *S. nigrum*, and the non-host, *N. attenuata*. *Pseudomonas thivervalensis* DR5 colonized roots of both *N. attenuata* and *S. nigrum* (1.4×10^9^ and 1.0×10^8^ cfu gFM^−1^, respectively). However, *P. thivervalensis* DR5 significantly decreased root length of *N. attenuata*, while increasing root length in *S. nigrum*. In addition, endophytes may have evolved from parasites and may still have parasitic tendencies [39] potentially contributing to incompatible interactions with non-hosts. *N. attenuata* may recognize the endophytic bacteria from *S. nigrum* as pathogens regardless of their stimulatory or inhibitory effects on *S. nigrum*. Root growth diminishes when energy is allocated for defense or for saving storage above-ground. Our observations of increased hypocotyl growth of *N. attenuata* upon inoculation with the selected endophytic bacteria isolated from roots of *S. nigrum* are consistent with such a scenario (Fig. 4B). *S. nigrum*, however, has likely evolved to be able to discriminate between its specific endophytes and pathogens thanks to its long association with its natural endophytic bacterial communities. The way in which *N. attenuata* copes with the endophytic bacteria in its roots appears to be different but has not yet been analyzed. When the two plant species were inoculated with “generalistic” PGP rhizobacteria from the DSMZ culture collection, their growth and fitness differed (Fig. 5A&B). Clearly, the PGP effects of natural endophytic bacteria on their host and non-host plant species are not the same.

Different behaviour of endophytic bacteria in the host and non-host plant species might be linked to the different environmental conditions under which the host and non-host grow. Black nightshades occur throughout the world in pioneer communities on open, disturbed and nutrient-rich soils, such as riverbanks, and have invaded many agricultural habitats, such as fields, gardens, and wasteland [19]. In contrast, *N. attenuata* evolved to optimize its growth in the immediate post-fire environment of deserts in southwestern United States; seeds germinate synchronously into nitrogen (N)-rich soils and hence have selected to grow rapidly when water availability is high [40]. Habitat-dependent co-evolution is likely to shape the particular endophytic bacterial communities that best fit a given habitat.

These findings demonstrate that natural endophytic bacteria with PGP traits do not have general and predictable effects on the growth and fitness of all host plants, although the underlying mechanisms are conserved. Clearly much more can be learned from studying interactions between natural endophytic bacteria and other native plant species in their ecological context.

## Supporting Information

Figure S1 Effects of exogenous IAA application on root growth of S. nigrum seedlings. Asterisks indicate significant differences (Fisher's PLSD test; P<0.05 (*) and P<0.0001 (***)).
(1.00 MB TIF)

Table S1 Seedling root colonization by endophytic bacterial isolates from S. nigrum. Bacterial re-isolation from seedling roots 7 days after inoculation with each bacterial isolate.
(0.03 MB DOC)
