# Supplementary figures and images for: Native Bacterial Endophytes Promote Host Growth in a Species-Specific Manner; Phytohormone Manipulations Do Not Result in Common Growth Responses
Source: PLoS One. 2008 Jul 16;3(7):e2702. doi: 10.1371/journal.pone.0002702 (PMC2444036; doi:10.1371/journal.pone.0002702)

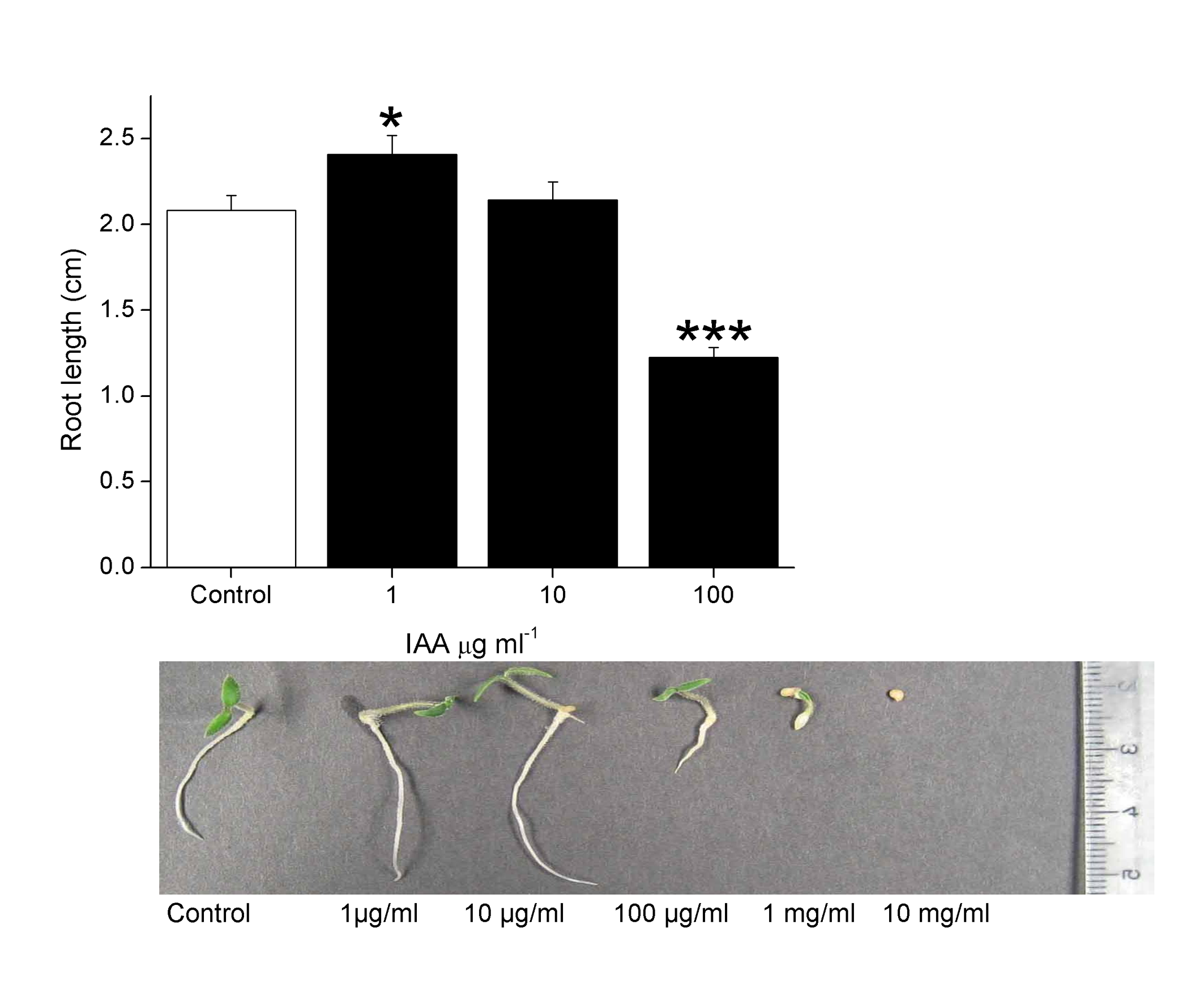

Supplement: Figure S1 — Effects of exogenous IAA application on root growth of S. nigrum seedlings. Asterisks indicate significant differences (Fisher's PLSD test; P<0.05 (*) and P<0.0001 (***)). (1.00 MB TIF) [file pone.0002702.s001.tif]
