# Supplementary material for: Native Bacterial Endophytes Promote Host Growth in a Species-Specific Manner; Phytohormone Manipulations Do Not Result in Common Growth Responses
Source: PLoS One. 2008 Jul 16;3(7):e2702. doi: 10.1371/journal.pone.0002702 (PMC2444036; doi:10.1371/journal.pone.0002702)
Supplement: Table S1 — Seedling root colonization by endophytic bacterial isolates from S. nigrum. Bacterial re-isolation from seedling roots 7 days after inoculation with each bacterial isolate. (0.03 MB DOC) [file pone.0002702.s002.doc]

**Table S1.**

| **Sample** | **Seedling colonization (cfu gFM^-1^)** |
| --- | --- |
| SSR4 | 2.3x10^6^ |
| SSR5-1 | 9.8x10^5^ |
| SSR5-2 | 4.0x10^6^ |
| SSR8-1 | 4.8x10^6^ |
| SSR8-2 | 3.2x10^6^ |
| BGCR2-8(1) | 3.3x10^6^ |
| DR5 | 3.2x10^6^ |
| BGCR2-6 | 4.4x10^6^ |
